# How to Keep Well

**Published:** 1912-09-15

**Authors:** W. A. Evans


### HOW TO KEEP WELL.

BY DR. W. A. EVANS.

The tissues of the human body are divided into hard workers, such as brain, kidney, and muscle, and those which merely stand and serve, such as bone and fat.

Of the workers, the muscles comprise the largest part. Their weight is sixty-five per cent of the body weight, after the bone weight has been subtracted. They do more than sixty-five per cent of the work. The brain sleeps, but the heart and breathing muscles work on through the resting hours.

The most important reason for eating is that the muscles may have food; for breathing, that they may have air.

If the muscles are burning up food properly, the stomach will take care of itself.

The real reason of dyspepsia and indigestion is in the muscles. The place where food is burned into waste is not in the kidneys or the liver but in the muscles. The kidneys and the liver merely eliminate the ashes as the lungs eliminate the smoke.

Muscle movement squeezes juices, rich in waste products, out of the tissue spaces and into the blood vessels, where circulation is more active.

Flabby, soft muscles mean several things. In the first place, they mean that sixty-five per cent of the man is not much man—is in a poor state of vigor. Besides that, they mean poor breathing, poor burning up of waste, clogged sewers all over his body, a certain amount of lowered resistance, and premature senility.

Every man needs muscular exercise. No man's work gives all his muscles the needed exercise. About nine-tenths of men are engaged in work requiring physical labor, yet these, too, require some form of play to exercise muscles unused in their work, to empty the dead ends of their sewers—for such are inactive muscles.

Properly planned, play, after the days' work is done, is restful to the mind and the body. As to housewives, brain workers, clerks, and office workers, there is no need of arguing that their muscles are flabby, their circulation poor, their digestion troublesome, their biliousness frequent, and that these call for better muscular development.

Every flabby person is willing to admit that he ought to take more exercise, but systems take time and apparatus. Some of them would build up their muscles if it could be done without apparatus or without going to a gymnasium.

Let them get busy, for to build up muscles it is not necessary to have apparatus or to go to any gymnasium or to take lessons from any physical culture professor, or to use up any particular time. One can be a home made Sandow.

The plan, which has been proposed any number of times, is that, instead of lifting a weight or chinning a bar, the lifting be done against opposing muscles. Nowhere in the body is there a group of muscles that works unopposed. Universally, whenever there is a group of muscles that pull in one direction, there is another group which pull opposite. The simplest and best plan to develop the muscels is to use the pull of one group against the other.

The man who is exercising according to this plan, should go over his body systematically at least once every day.

Step one is to pick out, in the mind, a group of muscles to be exercised. Step two is to move the part selected by pulling on one group of muscles and pulling against that group with the opposing group. Repeat this three times.

For this it is not necessary to know the names of the muscles. The motion having been decided on, experience teaches just what muscles are to be used to accomplish it.

Having gone over the face muscles, next go over those of the neck. For instance, draw the head back, pulling it violently with the muscles of the back of the neck, and pulling violently against these with the muscles of the front. Exercise all the muscles of the neck by pulling hard and slowly with each group opposed by a hard pull from each opposite.

The hands, writs, forearms and arms, legs, chest, back, and abdominal muscles are gone over in the same systematic way.

Five minutes in the bathroom in the morning, while one dresses, and five minutes as he undresses at night, will be all that is needed for the home part of the plan.

Following it, within a week, one can pick out any muscle and exercise it, and one can get as much squeezing out of waste from his biceps by bending his elbow with the triceps pulling opposite as he can by lifting a bag of shot.

The best part of the plan, however, is that as soon as one gets going he does not have to bend his limbs to get the exercise or to do anything else that attracts attention. As one rides in the street car or automobile, sits at his desk, or does anything else which does not occupy all his mental energy, he can throw his muscles into tension without moving or giving any outward sign.

Let the man who goes home from work in the afternoon loggy and heavy—perhaps suffering from headache—systematically pick out and contract all the muscles of his abdomen and back. He will not only gain for himself a muscle control that will keep his belly flat but he will flush out the waste of peripheral tissue, cause himself to breathe deeper, to think clearly, and better resist infection.

Following this very simple plan for ten minutes in the bathroom at home and from time to time during the day a man can build up muscle lumps that will compare favorably with the pictures in the street cars.

If the advice were stopped at this point this article would do more harm than good. Muscle building is not enough. Muscle training is of importance. A muscle building system must be supplemented by a muscle training system, and this last calls for play of some sort.

The big muscled fellows are not in very good condition. Many of them die from consumption and pneumonia. They do not live long; they never are good prize fighters or wrestlers; they never win any running races; they are never golf champions; they are never good billiard players nor bowlers; nor do they win any games at tennis.

Every one needs some play out of doors. There is genuine physical need of the freshness of the open air, in sunshine, and in storm. One needs difference in temperature—to breathe deeply of fresh air—to have the wind and rain beat in the face—to have the heart beat fast—to have the skin red from blood in the capillaries—to sweat.

Add to the above program for "auto-muscular" development some exercise in the open air, such as walking to and from the office or even climbing stairs instead of using the elevators.

Striking a golf ball is a wonderful art. For at least fifty muscles are required. Each must do just exactly its work, no more, no less. It must do its work at just exactly the right time—neither too soon or too late. Each must do its work in proportion to the work done by each other member of the "force." If any one lags or fails to do its share, some other must compensate in one way or another. —*Chicago Tribune.*

